# Markedly divergent effects of Ouabain on a Temozolomide-resistant (T98G) vs. a Temozolomide-sensitive (LN229) Glioblastoma cell line

**DOI:** 10.1007/s12672-023-00633-2

**Published:** 2023-02-25

**Authors:** Heidrun Weidemann, Daniel Feger, Jan E. Ehlert, Marcus M. Menger, Robert C. Krempien

**Affiliations:** 1 Clinic for Radiotherapy, HELIOS Hospital Berlin-Buch, Schwanebecker Chaussee 50, 13125 Berlin, Germany; 2 Reaction Biology Europe GmbH, Engesserstr.4, 79108 Freiburg, Germany; 3 Fraunhofer Institute for Cell Therapy and Immunology, Branch Bioanalytics and Bioprocesses (IZI-BB), Am Mühlenberg13, 14476 Potsdam, Germany

**Keywords:** Bcl-2, Cell migration, Chemoresistance, Glioblastoma, Na/K-ATPase, Ouabain, Pan-Akt, Plasma cell membrane potential

## Abstract

**Background:**

Glioblastoma multiforme (GBM) is the most aggressive primary brain tumor with poor prognosis. GMB are highly recurrent mainly because of radio- and chemoresistance. Radiotherapy with Temozolomide (TMZ) is until today the golden standard adjuvant therapy, however, the optimal treatment of recurrent glioblastoma remains controversial. Ouabain belongs to the Cardiotonic Steroids (CTS) the natural ligands of the Na/K-ATPase (NKA). It is established that the NKA represents a signal transducer with either stimulating or inhibiting cell growth, apoptosis, migration and angiogenesis. Over the last decade evidence grew that CTS have anti-tumor properties especially in GBM.

**Aim:**

Proceeding from recent studies we wanted to further demonstrate a divergent effect of Ouabain on a TMZ-resistant (T98G) as compared to a TMZ-sensitive (LN229) GBM cell line.

**Methods:**

We analyzed the effect of Ouabain on cell migration and plasma cell membrane potential (PCMP) in the LN229 and T98G GBM cell line as well as underlying mechanisms (Bcl-2 and p-Akt/pan-Akt expression). Moreover, we analyzed the anti-angiogenic effect of Ouabain on human umbilical vein endothelial cells (HUVECs).

**Results:**

T98G cells showed a significant inhibition of cell migration and a significant depolarization of the PCMP at similar Ouabain concentrations (IC50 = 1.67 × 10^–7^ M) resp. (IC50 = 2.72 × 10^–7^ M) with a strong inverse correlation (R^2^ = 0.95). In contrast, LN229 cells did not respond to Ouabain in these assays at all. Similarly, only T98G but not LN229 cells revealed Bcl-2 down-regulation at nanomolar Ouabain concentrations. This unique response to Ouabain is associated with a down-regulation of pan-Akt in T98G cells 24 h after Ouabain (1.0 × 10^–6^ M) treatment. For the first time, the anti-angiogenic effect of Ouabain on HUVEC cells (IC50 = 5.49 × 10^–8^ M) was demonstrated which correlated strongly with the anti-migratory effect (R^2^ = 0.85).

**Conclusion:**

The TMZ-resistant T98G cell line as compared to the TMZ-sensitive LN229 cell line shows a high sensitivity towards Ouabain. We consider it as a promising new compound especially in recurrent GBM to overcome the resistance to TMZ and irradiation.

## Introduction

Glioblastoma multiforme (GBM) is considered the most aggressive, invasive and undifferentiated type of gliomas and has been designated Grade IV by the World Health Organization (WHO). Gliomas are conditioning their microenvironment by priming glioma-associated microglia and macrophages (GAMs) to generate an immunosuppressed niche for tumor expansion [1]. They are characterized by a specific diffuse infiltrative growth and high vascularization, which explains the extreme therapeutic challenge and high morbidity and mortality [2]. With the standard optimal treatment, the median survival does not yet exceed 12 to 15 months for patients with high-grade (WHO IV) glioblastomas [3].

Radiotherapy is crucial in the adjuvant setting of malignant gliomas. The pitfall is that 90% of the tumors recur at the primary location [4]. One important reason is the development of resistance towards radiotherapy [5], mainly due to the survival of cancer stem cells in hypoxic microenvironment niches of the tumor core [6, 7]. Chemotherapy slowly gains more importance in the treatment of malignant gliomas. Temozolomide (TMZ) belongs to the alkylating agents, i.e., by transferring methyl groups to guanin bases of DNA, the replication is impaired leading to double strand breaks and apoptosis. The responsible main factor for TMZ resistance is a high level of O6-methylguanine methyltransferase (MGMT) which can repair methylated DNA. It was shown that the 2 years overall survival rate (OSR) was significantly higher with TMZ compared to radiotherapy alone (26.5% vs. 10.4%) [8]. These data established radiotherapy with concomitant Temozolomide as golden standard adjuvant therapy for newly diagnosed glioblastomas [8, 9]. An intensive research is ongoing to identify new chemotherapeutic combinations [10–12], targeted molecular [13–15] and antiangiogenic agents [16, 17] which may overcome the resistance especially towards Temozolomide [18]. To mention here the specific role of autophagy as target in GBM therapy [19]. Also in the field of immunotherapy [20] and bionanotechnology [21] enormous efforts are currently made to develop an “ideal drug” for gliomas.

The Cardiotonic Steroids (CTS) constitute a family of steroid hormones which decades ago were discovered in plants e.g., Ouabain from an African tree (Acokanthera ouabaio). It is nowadays established that Ouabain is synthesized and released mainly in the adrenal gland as demonstrated in different species [22–24]. Moreover it was shown that, like other “stress hormones”, they are part of the Hypothalamic–Pituitary–Adrenal axis and stimulated by the adrenocorticotropic hormone (ACTH) and Angiotensin II [25, 26]. There is still controversy whether endogenous Ouabain (EO) is identic to authentic Ouabain [27, 28] due to methodic difficulties in purification and detection of EO. Therefore, a simple diagnostic tool for steroid glycosides like Ouabain by specific molecular recognition elements like antibodies or aptamers would be very desirable for further studies. Interestingly, highly specific aptamers against several steroids, like the glycoside digoxin, have recently been developed [29, 30].

The CTS bind to the sodium pump Na/K-ATPase (NKA), whose classical function is to create an electrochemical gradient across the plasma cell membrane (PCM) hereby providing the intracellular milieu for important electrical and biochemical processes. The NKA consists of the alpha unit (with four isoforms α1—α4), the beta unit (with 3 isoforms, β1–β3) and, in some tissues, seven members of the FXYD protein family [31]. Interestingly, not only each organ but also many cancers (bladder, prostate, gastric) show unique isoform patterns e.g., reduced expression of the α1 isoform and increased expression of the α3 isoform [32–34]. The NKA α1 isoform is responsible not only for cell proliferation but also for high intracellular gluthathione levels that prevent reactive oxygen species (ROS) induced apoptosis [35]. Nowadays, there is high evidence that the NKA represents also a signal transducer and, remarkably, the induction of many signaling pathways is partly independent from its pump activity [36–38]. Since their first discovery as potential anti-cancer drugs half a century ago [39], in the last decade the CTS gained increasing attention as anti-tumor compounds [35, 40–43].

Recently, interest shifted to the interference of CTS with Glioblastoma multiforme. Badr and coworkers revealed in a huge drug screening trial (comprised of more than 1000 bioactive compounds) that the CTS enhanced the sensitivity of glioblastoma multiforme cells to tumor necrosis factor-related apoptosis-inducing ligand (TRAIL)-induced apoptosis [44]. Denicolai and coworkers identified the cardiac glycosides Proscillaridin A and Ouabain as the most effective compounds on glioblastoma cells (Prestwick chemical library® with more than 1100 molecules). Proscillaridin A impaired GBM stem cell self-renewal capacity even at relative low range (0.05–0.1 µM) [45]. Chen and coworkers demonstrated in two Glioblastoma cell lines, LN229 and T98G that the inhibition of NKA by Ouabain (0.1–10 µM) induced “hybrid cell death”, a mixture of apoptosis and necrosis, which enhanced their sensitivity to Temozolomide. Remarkably, Ouabain selectively killed TMZ-resistant T98G cells at low concentrations (0.1 µM) [46]. Cheng and coworkers observed in the GBM cell line U-87MG that the suppression of motility and migration after treatment with Ouabain (2.5 to 25 µmol/l) correlated with a down-regulation of phosphorylated Akt (p-Akt) also called protein kinase B (PKB), hypoxia-inducible factor 1-alpha (HIF-1α) and phosphorylated mammalian target of rapamycin (p-mTOR) [47].

In summary, there is growing evidence that Ouabain (and other CTS) have promising anti-cancer effects and might increase and/or restore chemo- and radiosensitivity of GBM cells [48–50]. Unfortunately, there was no real progress in recent years to establish them in clinical settings for different assumed reasons. First, all CTS are known (from their clinical use in cardiology) to have a narrow therapeutic window. At this point it must be mentioned that in many in vitro studies (see above) the effective range of Ouabain in human cancer cell lines was 0.1–1 µM which is about two to three orders of magnitude higher than the therapeutic range of CTS (1–50 nM) in humans [51, 52] and the human endogenous Ouabain plasma concentrations (10–1000 pM) [53–55]. We will discuss this important issue later.

Second, the effects of CTS are very fine-tuned in a dose- and time-dependent matter i.e., even in one cell type they can induce cell proliferation, cell differentiation, apoptosis, and necrosis. This principle applies to benign as well as malignant cells.

Interestingly, a kind of “double duality” of CTS i.e., their dose-dependency and their different effects on malignant vs. benign cells could turn out to be advantageous for clinical applications in oncology. Malignant cells obviously are more sensitive to CTS/Ouabain i.e., at low concentrations normal cells proliferate, whereas malignant cells may already undergo apoptosis (“Lower-Threshold-theory” by Weidemann and coworkers) [56].

In recent years, with increasing evidence of the paramount role of ion channels and the plasma cell membrane potential (PCMP) in tumorigenesis and tumor cell migration the CTS might undergo a “revival” especially in the treatment of GBM [57–59]. Ion channels have multiple functions e.g., they modify the cell cycle checkpoints and thereby support cell proliferation. Several studies pointed to the importance of cell membrane hyperpolarization, due to K + channel activation [49] to enable the passage of cells through G0/G1 or the G1/S phase [60]. Knox and Gilbert [61] revealed that hyperpolarization of the cell membrane is associated with increased radioresistance and that increased expression of the B-cell lymphoma 2 (Bcl-2) gene is associated with membrane hyperpolarization [61]. They assumed that Bcl-2 interacts at the inner plasma cell membrane with the NKA as the ubiquitous natural modulator of the PCMP. When they treated Bcl-2 transfected PW lymphoma cells with low dose (1 µM) Ouabain known to inhibit the NKA without inducing itself programmed cell death the protection from radiation-induced apoptosis disappeared [62]. Interestingly, this assumption—the interaction of Bcl-2 with Ouabain/NKA—recently has been proven by Lauf and coworkers [63].

**Aim:** Proceeding from the interaction of Ouabain with NKA in Glioblastoma cells and the hypothesized significance of the plasma cell membrane potential with respect to GBM migratory capacity and treatment resistance, we think that it is reasonable and of therapeutic value to further analyze the effect of Ouabain on GBM cells. We chose specifically the TMZ-sensitive LN229 and the TMZ-resistant T98G cell lines to demonstrate the assumed divergent effects of Ouabain on cell migration, cell membrane potential as well as Bcl-2 expression and to confirm its inhibiting function on angiogenesis. Furthermore, we aimed to reveal some underlying mechanisms i.e., p-Akt/pan-Akt expression.

## Materials and methods

### Cell culture

The human glioblastoma cell lines LN229 and T98G (LGC/ATCC, Wesel, Germany) were cultured in Dulbecco's Modified Eagle Medium (DMEM) with GlutaMAX™ Supplement (Fisher Scientific GmbH, Schwerte, Germany) containing high (4.5 g/L) glucose, no pyruvate, 10% FBS, and 1% Antibiotics. Primary human umbilical vein endothelial cells (HUVECs) (Promocell, Heidelberg, Germany) were cultured in endothelial growth medium (Promocell) containing 10% FBS and 1% antibiotics.

### Chemicals, proteins

All chemicals were purchased from Selleckchem (USA). The Na^+^/K^+^ ATPase inhibitor Ouabain was completely dissolved in dimethyl sulfoxide (DMSO) as a stock solution of 10 mM. 1000 × concentrated solutions in DMSO were prepared for each assay type, except for the angiogenesis assay, where 100 × concentrated solutions were used. All assay conditions including the solvent controls were normalized to equal final concentrations of DMSO. For migration assay, the compound SKI-606 (Bosutinib) was used as reference compound. It is a very potent inhibitor of Src family kinases causing a decrease in cell motility [64]. It was demonstrated that cell proliferation was not affected at concentrations needed to inhibit cell migration [64]. Sunitinib, a multi-targeted receptor tyrosine kinase inhibitor potently targeting VEGF-R2, was used as reference compound in the angiogenesis assay. The proangiogenic ligand of VEGF-R2, human recombinant VEGF-A was provided by ProQinase GmbH (Freiburg, Germany). The viability dye Calcein-AM was purchased from Thermo Fisher Scientific (Waltham, MA, USA).

### Migration assay

Migration assay was done using Collagen I-precoated ORIS-96 well plates (AmsBio, USA) according to the manual. Briefly, cells were seeded in 150 µl growth medium on ORIS plates. After 24 h, stopper inserts were removed except for the zero controls. Medium was exchanged and 10 µl of test samples containing Ouabain and the reference SKI-606 in a concentration range of 10^–12^ to 10^–5^ M were added to the cells. After a migration phase of 24 h, medium was substituted with 75 µL DMEM w/o Phenol red containing 2 µg/ml Calcein-AM. Cells were incubated for 15 min at 37 °C and finally, fluorescent cells in the insert-defined area were detected by a fluorescence microplate reader (Fluostar, BMG, Germany) using FITC-settings (EX/EM = 485/520 nm). Subsequently fluorescence photographs of each well were taken at 40 × magnification. Each condition was done in duplicate.

### Cell membrane potential

The Cellular Membrane Potential Assay Kit (Abcam, Germany) was used according to the manual. Briefly, cells were plated in 100 µl growth medium on a black 96 well cell culture plate (Corning, USA) overnight. Subsequently, 100 µl of the MP dye-loading solution was added to the cells. The 96 well plate was incubated for 30 min at 37 °C and 5% CO_2_. Before adding the compounds, the fluorescence signal was measured at EX/EM = 540/590 nm using a Fluostar plate reader (BMG, Germany). Afterwards, cells were treated with a deca-log dilution series of Ouabain using a Tecan nano drop dispenser (Tecan, Switzerland). Each condition was done in duplicate. As control, cells were treated with the solvent DMSO. After 2 h, 4 h, and 6 h, fluorescence was measured from the bottom of the wells.

### Bcl-2 expression

For the analysis of the Bcl-2 expression the human Bcl-2 ELISA Kit (Abcam, Germany) was used. Cells were seeded in 1 ml growth medium on a 12 well cell culture plate (Corning) and incubated at 37 °C overnight. The next day compounds were added to the cells. As vehicle control DMSO was used. After a treatment of 24 h, cell supernatants were collected, cells were harvested by scraping in PBS and added to the corresponding supernatant. Subsequently, cells were washed once in PBS, and harvested by centrifugation. PBS was aspirated and cells were re-suspended in 100 µl of 1 × lysis buffer from the ELISA kit, incubated for 60 min at 37 °C with gentle shaking. Cell lysates were centrifuged at 1000 × g for 15 min and cleared supernatants were collected and stored at − 80 °C. Detection of Bcl-2 protein was done according to the instructions in the manual of the kit. Briefly, all samples and standard solutions were equilibrated to room temperature, the pre-coated microplate was washed twice, standards and samples (1:5 diluted in sample diluent) were added and incubated with biotin-conjugated antibody for 2 h. After three wash steps Streptavidin-HRP was added and incubated for 1 h. The plate was again washed 3 times and incubated with TMB Substrate for 10 min before stop solution was added. Subsequently, the optical density (OD) values at 450 nm and 540 nm were measured. The OD_540_ values were subtracted as background from the OD_450_ values. Each condition was done in duplicate.

### Angiogenesis assay

Spheroids of primary HUVEC cells embedded in a collagen matrix represent a robust in vitro model to analyze the pro- or antiangiogenic potential of compounds in preclinical studies [65]. The experiments were pursued in modification of the originally published protocol [66]. In brief, spheroids were prepared as described by Korff and Augustin [67] by pipetting 400 HUVECs in a hanging drop on plastic dishes to allow overnight spheroid aggregation. Fifty HUVEC spheroids were then seeded in 0.9 ml of a collagen gel and pipetted into individual wells of a 24 well plate to allow polymerization. After 30 min 100 µl of test compounds mixed with the growth factor VEGF-A were added on top of the polymerized gel. Plates were incubated at 37 °C for 24 h and fixed by adding 4% PFA (Roth, Karlsruhe, Germany). The sprouting intensity of HUVEC spheroids treated with the test samples were quantified by an image analysis system determining the cumulative sprout length (CSL) per spheroid. Pictures of single spheroids were taken using an inverted microscope and the digital imaging software NIS-Elements BR 3.0 (Nikon, Germany). Subsequently, the spheroid pictures were uploaded to the homepage www.wimasis.com of the company Wimasis (Wimasis, Cordoba, Spain) for image analysis. The cumulative sprout length of each spheroid was determined using the imaging analysis software tool WimSprout (Wimasis, Cordoba, Spain). The mean of the cumulative sprout length of 10 randomly selected spheroids was analyzed as an individual data point.

### Pan-Akt and p-Akt ELISA

Detection of pan-Akt and p-Akt-Ser473 levels after cell treatment was done using the AKT1/2/3 (Total/Phosphor) InstantOne ELISA Kit from Thermo Fisher Scientific (Waltham, MA, USA). For treatment, cells were seeded in growth medium on a 96 well cell culture plate and incubated at 37 °C overnight. The next day compound was added to the cells. After a treatment of 24 h, supernatants were removed, and cells were lysed by adding freshly prepared 1 × Cell Lysis Buffer Mix from the kit and subsequently shaking at 300 rpm for 10 min. Lysates were further processed according to the kit manual. Briefly, 50 µl of lysates were added to the assay plate. The same was done with a 1:2 dilution series of the kit positive control. Wells with lysis buffer only served as negative control. Adequate antibody cocktails for pan-Akt or p-Akt detection were added to the test samples and incubated for 2 h at room temperature. Subsequently, wells were washed twice 3 times with PBS/0.1% (v/v) Tween-20 using a 96 well plate washer. After completely removing the wash buffer by inverting the plate on a paper towel, detection reagent was added. The color development was stopped by adding stop solution and the absorbance was measured at 450 nm and 540 nm using a plate reader. The OD_540_ values were subtracted as background from the OD_450_ values. For data analysis, the mean value of the negative control was subtracted from each OD_450_ value. Levels of pan-Akt and p-AKT were converted into percentage of the corresponding vehicle control, which was set to 100%. This was separately done for each cell line and each target. Each condition was done in duplicate.

### Statistics

All statistics were performed by OriginPro 2021 software (OriginLab Corporation, Northampton, MA, USA), e.g., the IC_50_ values were estimated by a nonlinear regression analysis, fitting the data to a dose–response function by the Levenberg Marquardt iteration algorithm, and the R^2^ values of the correlation analysis were estimated by a simple linear regression. Additionally, the statistically significance was determined by Welch’s unequal variances t-test and the level of significance is termed by the p-values (*p < 0.05 and **p < 0.01).

## Results

### Cell migration

In order to evaluate the effect of Ouabain on cell motility and migration, one of the most aggressive characteristics of GBM, we chose a wound healing assay with a cell-permeable dye. LN229 and T98G cells were treated for 24 h with either Ouabain or the reference compound SKI-606 in a concentration range of 10^–12^ to 10^–5^ M, the negative control was the solvent DMSO.

**Fig. 1:**
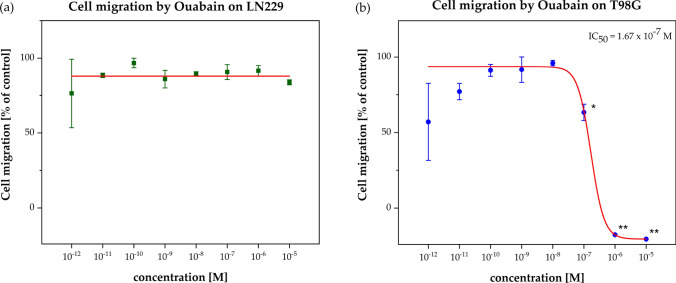
Cell migration assay with cell line LN229 (**a**) and T98G (**b**). The cell migration (% of control) was plotted as the function of the Ouabain concentration (10^–12^ to 10^–5^ M). The red curves represent the nonlinear regression analysis for the IC_50_ calculation. The inhibition of cell migration was only observed for the TMZ-resistant cell line T98G by IC_50_ = 1.67 × 10^–7^ M. Error bars show the range of measured values of each duplicate and the level of significance is shown by the p-value range (*p < 0.05 and **p < 0.01)

**Fig. 2:**
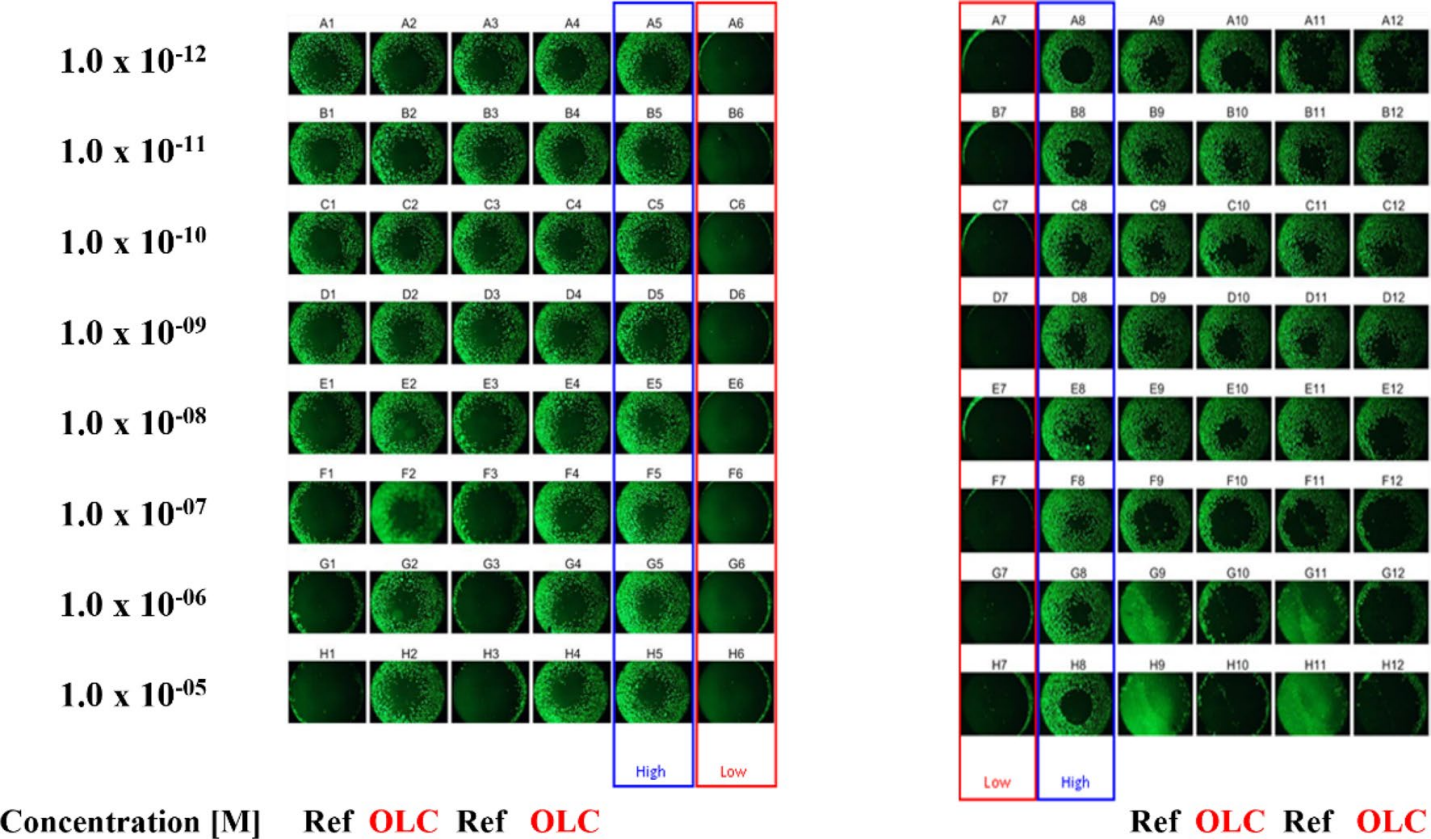
Detection of cell migration with cell-permeable dye. Calcein-AM labeled LN229 (left) and T98G (right) cells in the migration area of the 96 well ORIS plate were photographed with a magnification of 40 × using a Nicon Eclipse TE2000-U fluorescence microscope and the software NIS Elements 3.0. OLC is Ouabain and Ref is SKI-606. Median value of solvent control (DMSO) was set to 100% (high control – blue box) and median value of zero controls (with stopper inserts) was set to 0% (low control – red box). (Conc: from 1 × 10^–5^ M at bottom to 1 × 10^–12^ M at top)

The TMZ-sensitive cell line LN229 did not show any inhibition of cell migration in response to Ouabain (Figs. 1 and 2). In contrast, the cell migration of the TMZ-resistant T98G cell line was significantly (p < 0.05 and p < 0.01) inhibited by Ouabain (Figs. 1 and 2). Remarkably, the reaction started abruptly at IC_50_ of 1.67 × 10^–7^ M so that one might assume a cytotoxic effect. But microscopic analysis did not reveal any necrotic shapes of the cells at the migration front, indicating that there is no cytotoxic effect even at the highest (10^–5^ M) Ouabain concentrations.

### Cell membrane potential

In order to analyze changes in plasma cell membrane potential which is seen in many tumor cells prior to apoptosis we chose a fluorometric assay. LN229 and T98G cells were treated with Ouabain (10^–12^ to 10^–5^ M). As control, cells were treated with the solvent DMSO. After 2 h, 4 h and 6 h fluorescence was measured at the bottom of the wells.

**Fig. 3:**
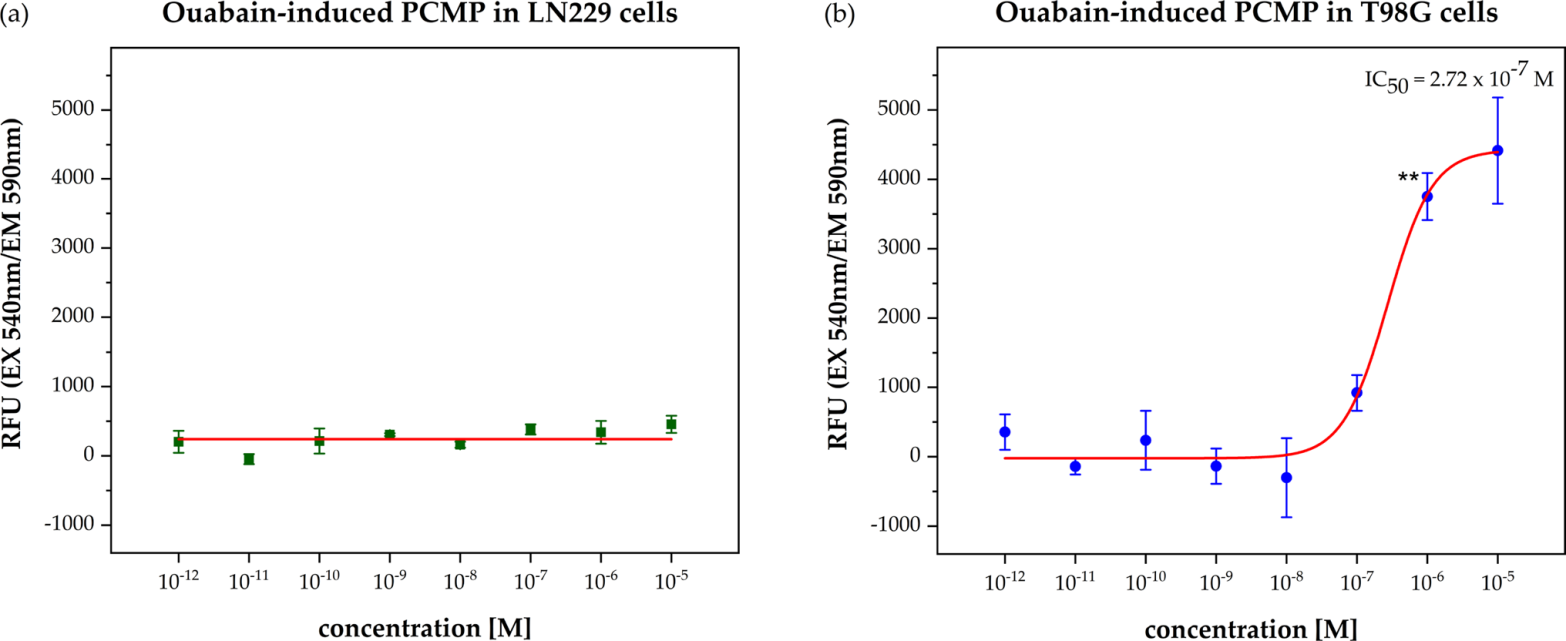
Plasma cell membrane potential (PCMP) assay with cell line LN229 (**a**) and T98G (**b**). The relative fluorescence units (RFU) were plotted as the function of the Ouabain concentration (10^–12^ to 10^–5^ M). Fluorescence values of the solvent control was defined as background and was subtracted from all single values. Results are shown after a treatment period of 4 h. The red curves represent the nonlinear regression analysis for the IC_50_ calculation. The significant concentration-dependent fluorescent change was only observed for the TMZ-resistant cell line T98G by IC_50_ = 2.72 × 10^–7^ M, which showed a clear depolarization of the plasma cell membrane. Error bars show the range of each duplicate and the level of significance is shown by the p-value range (**p < 0.01)

**Fig. 4:**
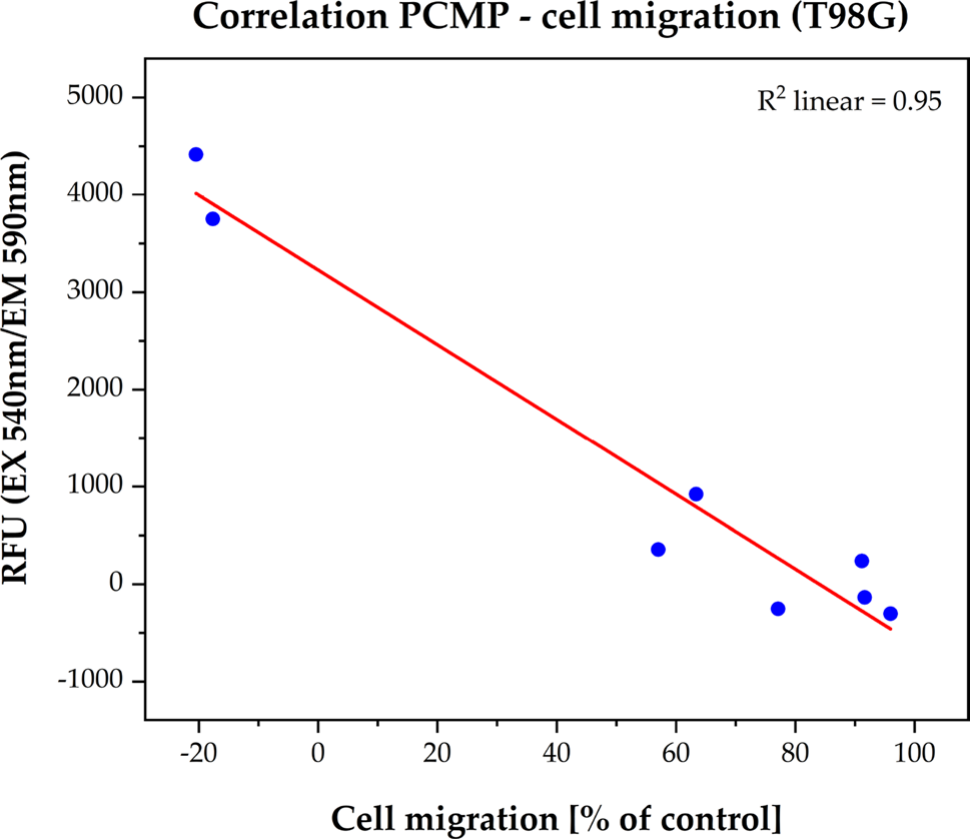
Correlation between plasma cell membrane potential and cell migration in the cell line T98G. The red curves represent the linear regression analysis for the Ouabain concentration (10^–12^ to 10^–5^ M) with the coefficient of determination (R^2^ = 0.95) based on Pearson correlation

The LN229 cells did not reveal any change in their plasma cell membrane potential (Fig. 3a). In contrast, the T98G cell line responded significantly (p < 0.01) with a clear depolarization of the plasma cell membrane after 4 h treatment with Ouabain at concentrations (IC_50_ = 2.72 × 10^–7^ M) which are in range of inhibiting the NKA (Fig. 3b). Interestingly, a significant inverse correlation (R^2^ = 0.95, p = 0.002) between plasma cell membrane potential and cell migration inhibition was seen (Fig. 4).

### Bcl-2 expression

In order to analyze the Bcl-expression which is known not only to have impact on apoptosis but also on the plasma cell membrane, a Bcl-2 ELISA kit was used. LN229 and T98G cells were treated for 24 h with Ouabain in a concentration range of 10^–12^ to 10^–5^ M, the negative control was DMSO.

**Fig. 5:**
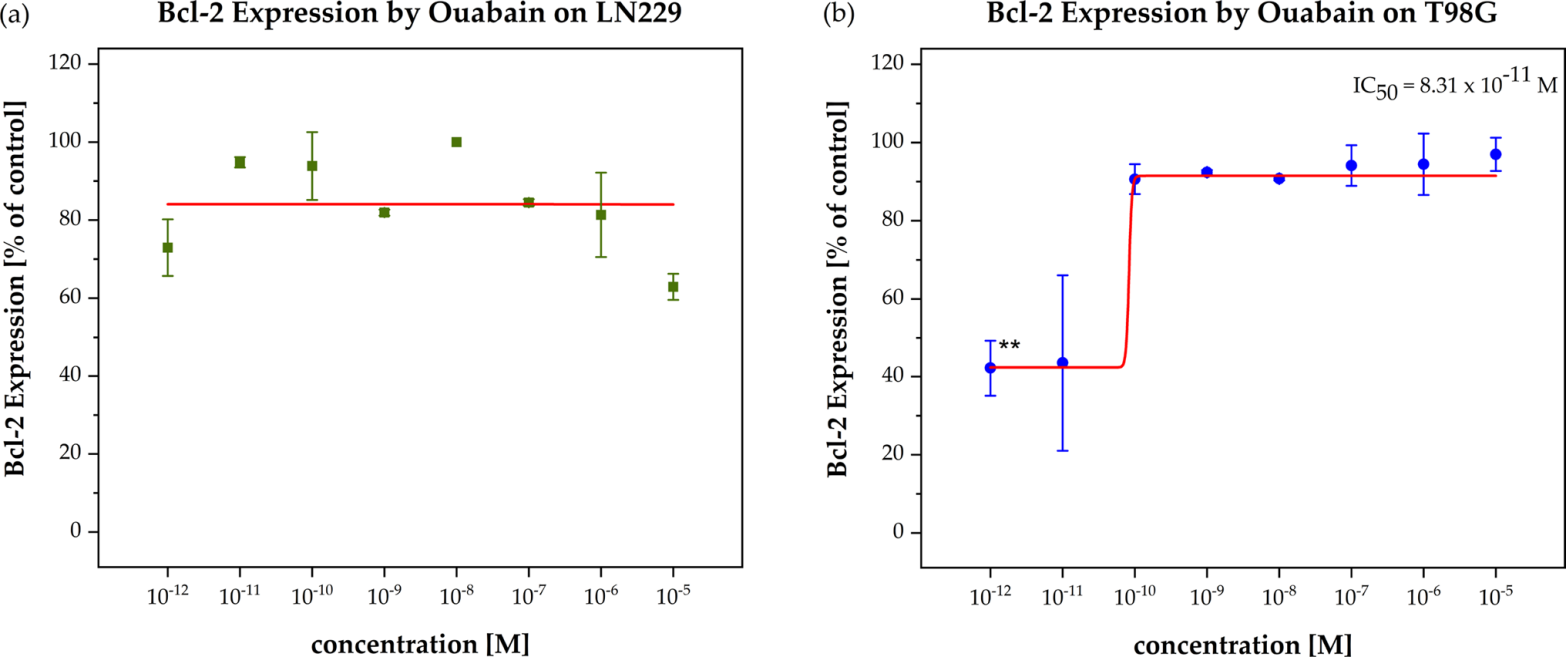
Bcl-2 Down-regulation of Bcl-2 expression in cell line LN229 (**a**) and T98G (**b**). The Bcl-2 expression (% of control) was plotted as the function of the Ouabain concentration (10^–12^ to 10^–5^ M) after 24 h treatment. OD450-540-values of cell lysates measured in 1:5 dilution in the Bcl-2 ELISA-kit are presented. The red curves represent the nonlinear regression analysis for the IC_50_ calculation. The down-regulation of Bcl-2 expression was only observed for the TMZ-resistant cell line T98G by IC_50_ = 8.31 × 10^–11^ M. Error bars show the range of measured values of each duplicate and the level of significance is shown by the p-value range (**p < 0.01)

With regard to the assumed down-regulation of Bcl-2 by Ouabain we did not see any effect of Ouabain on the LN229 cell line. In the T98G cells, however, a significant Bcl-down-regulation was detected, but only at very low Ouabain concentrations (IC_50_ = 8.31 × 10^–11^ M, p < 0.05). We will later discuss this phenomenon (Fig. 5).

### Angiogenesis

In order to analyze the effect of Ouabain on angiogenesis, for tumor cells a prerequisite for cell migration, we used a standard protocol with HUVEC spheroids. They were treated for 24 h with either Ouabain or the reference compound Sunitinib in a concentration range of 10^–12^ to 10^–5^ M mixed with VEGF-A.

**Fig. 6:**
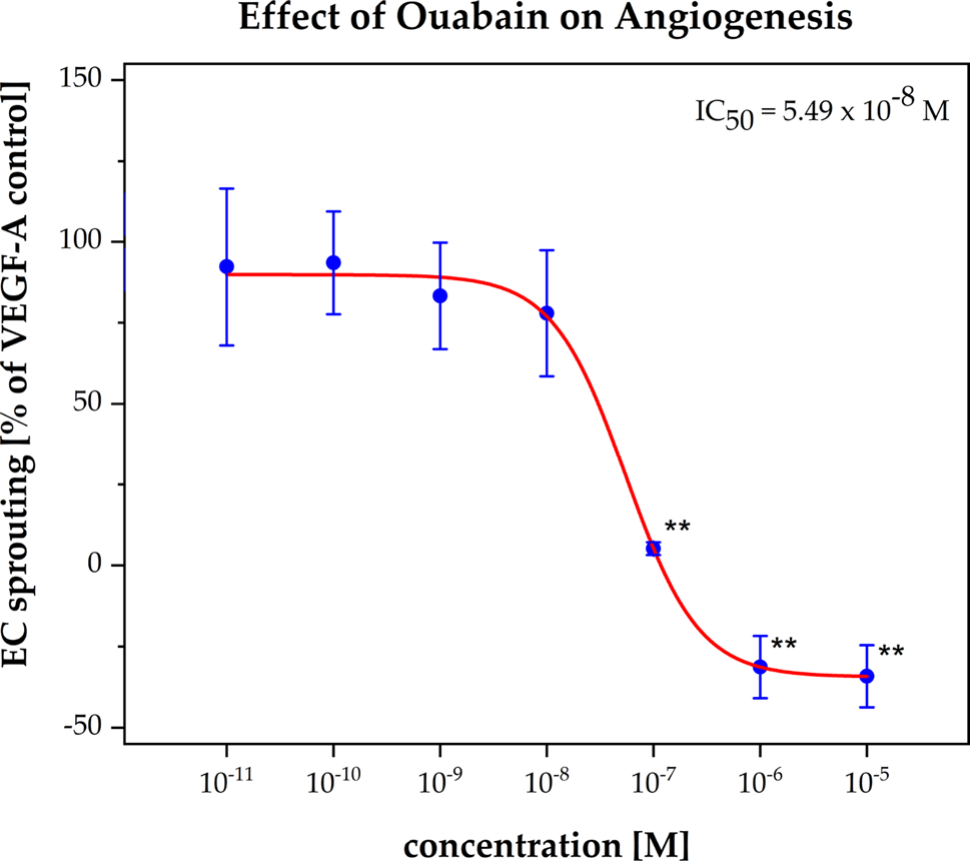
Effect of angiogenesis. The VEGF-A induced HUVEC spheroid sprouting (% of VEGF-A control) was plotted as the function of the Ouabain concentration (10^–12^ to 10^–5^ M). The red curves represent the nonlinear regression analysis for the IC_50_ calculation. The angiogenesis effect was observed by IC_50_ = 2.30 × 10^–6^ M. Error bars show the range of measured values of each duplicate and the level of significance is shown by the p-value range (*p < 0.05 and **p < 0.01)

**Fig. 7:**
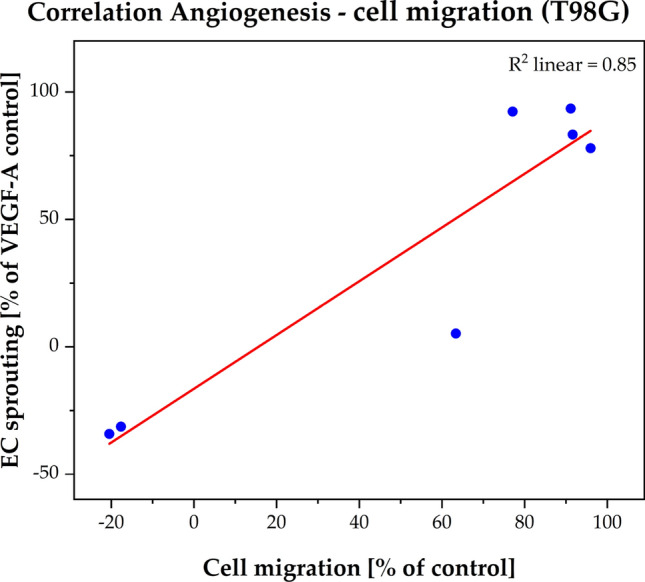
Correlation between angiogenesis and cell migration by the cell line T98G. The red curves represent the linear regression analysis for the Ouabain concentration (10^–12^ to 10^–5^ M) with the coefficient of determination (R^2^ = 0.85) based on Pearson correlation

We could demonstrate that Ouabain inhibited significantly (p < 0.05 and p < 0.01) angiogenesis (HUVEC) at similar (IC_50_ = 5.49 × 10^–8^ M) concentrations (Fig. 6) as needed to inhibit cell migration (IC_50_ = 1.67 × 10^–7^ M). Remarkably, we could demonstrate a strong positive correlation between inhibition of angiogenesis and inhibition of cell migration (Fig. 7).

### Akt/p-Akt expression

Finally, to analyze the effect of Ouabain on Akt-activation which is part of a crucial signaling pathway in cell migration, a dual ELISA kit was used. LN229 and T98G cells were treated for 24 h with Ouabain (10^–13^ to 10^–6^ M), the negative control was DMSO.

**Fig. 8:**
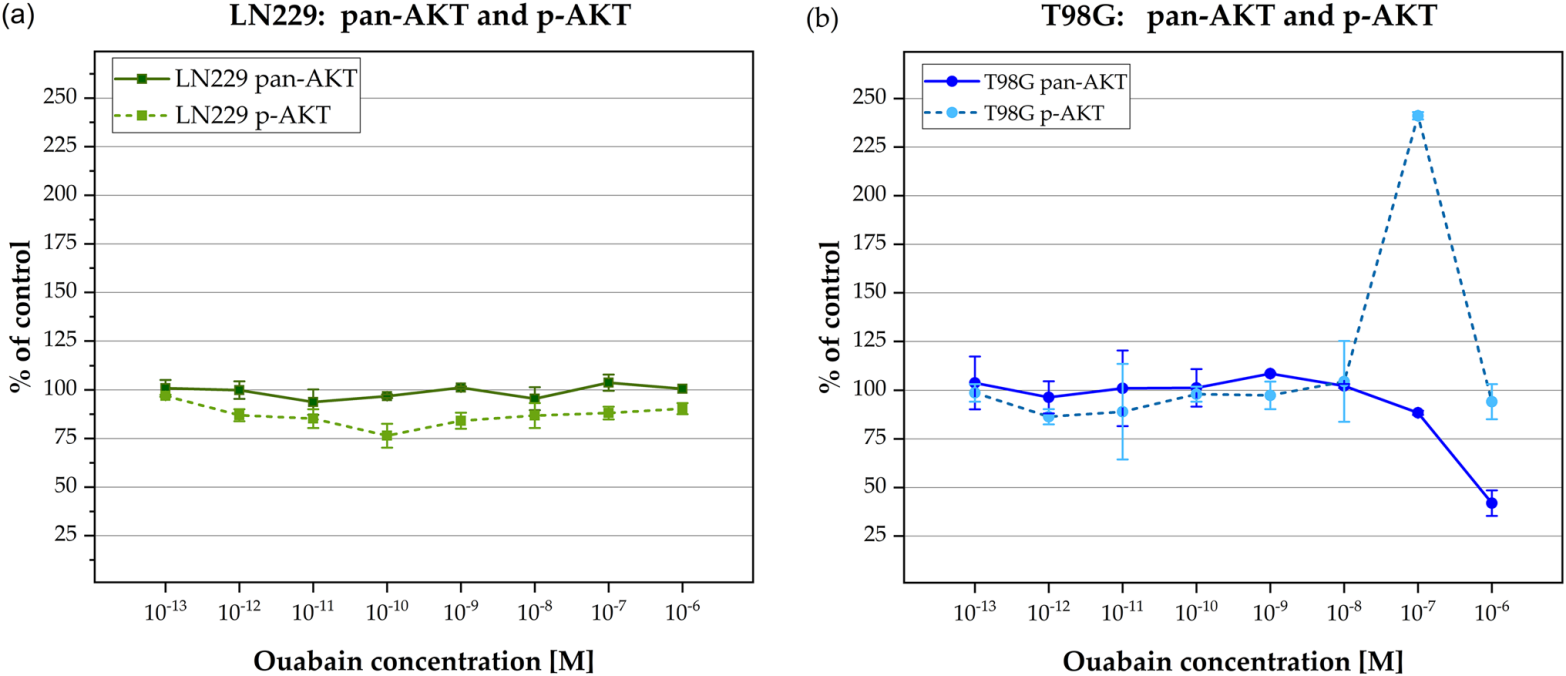
Akt activation in cell line LN229 (**a**) and T98G (**b**). The pan-Akt (dark points/line) and p-Akt (light points/dashed line) activation (% of control) was plotted as the function of the Ouabain concentration (10^–13^ to 10^–6^ M) after 24 h treatment. A significant change was only observed for the TMZ-resistant cell line T98G with an increase of p-Akt at 0.1 µM Ouabain and a decrease of pan-Akt at 1 µM. Error bars represent the range of measured values. OD_450-540_-values were presented in % of vehicle control (= 100%)

The LN229 cells did not show any change under Ouabain treatment neither in pan-Akt nor in p-Akt expression. In contrast, in T98G cells after 24 h Ouabain treatment a sharp up-regulation of p-Akt at 0.1 µM was seen as well as a significant down-regulation of pan-Akt at higher (1 µM) Ouabain concentration (Fig. 8).

## Discussion

The results show that with regard to cell migration as well as plasma cell membrane depolarization Ouabain indeed has different effects on the two GBM cell lines, the TMZ-sensitive LN229 and the TMZ-resistant T98G cell line, similar to the results found by the group of Chen and coworkers with respect to apoptosis [46]. The authors showed that in T98G cells apoptosis was induced at significantly lower Ouabain concentrations (0.1 μM) as compared to LN229 cells (> 1 µM). However, in our setting, LN229 cells did not show any reaction to Ouabain neither in the migration assay nor in the cell membrane potential assay, even at higher concentrations (> 10 µM). For this discrepancy we do not have a plausible explanation and further studies are needed.

In contrast, the TMZ-resistant cell line T98G showed a marked inhibition of migration at rather low doses of Ouabain (0.01–0.1 µM), which correlated significantly with an increase in cell membrane depolarization (p = 0.002). A similar opposite reaction pattern to Ouabain was observed in the LN229 and T98G cells in the Bcl-2 analysis i.e., only in the T98G cell line we saw a down-regulation whereas LN229 did not show any reaction. The fact, that the Bcl-2 down-regulation was detected only at very low Ouabain concentrations (0.01 nM) must be interpreted with caution, we will discuss this issue further down. In summary, while the TMZ-resistant T98G cell line is sensitive to Ouabain, the TMZ-sensitive LNN29 cell line seems to be resistant to Ouabain.

Interestingly, migrating GBM cells are per se resistant to apoptosis. Joy and coworkers revealed an activation of the phosphoinositide 3-kinase (PI3-K) survival pathway by migrating glioma cells, which renders them resistant to apoptosis [68]. Applying a specific inhibitor of PI3-K (LY294002) to migrating cells the phosphorylation of Akt was inhibited and consequently, an increased rate of apoptosis was seen. Yang and coworkers could demonstrate that Ouabain is able to prevent phosphorylation of Akt and mTOR, inhibiting cell migration and enhancing apoptosis [47]. Lefranc and coworkers also stressed the importance of an inverse relationship between migration and apoptosis in GBM and the key role of the PI3-K/Akt pathway [69].

The fact that the T98G cells reacted to the cell migration assay as well as to the plasma cell membrane potential assay at similar Ouabain concentrations strongly indicates a causal relationship between migration inhibition, depolarization of the cell membrane, and consequent induction of apoptosis.

Many authors described a correlation between plasma cell membrane (PCM) depolarization and early apoptosis, but it is not fully clear whether it constitutes a causal relationship or a mere epiphenomenon. There is evidence that PCM depolarization is a prerequisite for apoptosis. Suzuki-Karasaki and coworkers described the disruption of intracellular K + and Na + concentrations as a basic important event leading to depolarization, cell shrinkage, and hence apoptosis [70]. Bortner and coworkers reported in Jurkat T-cells a PCM depolarization immediately after application of diverse apoptotic stimuli (anti-Fas antibody, thapsigargin and the calcium ionophore A23187) followed by cell shrinkage [71]. Moreover, an early increase in intracellular Na + as well as inhibition of K + uptake was observed in response to anti-Fas, indicating an inactivation of the Na + /K + -ATPase. Interestingly, Ouabain enhanced anti-Fas-induced apoptosis. Finally, applying an anti-apoptotic signal, i.e., protein kinase C, did not only inhibit apoptosis but also prevent cell membrane depolarization in response to anti-Fas. Thus, the authors concluded that cell membrane depolarization per se is a crucial early step in anti-Fas-induced apoptosis [71].

Interestingly, the PCM depolarization was not a short-lasting phenomenon, as known from electrically excitable cells, but rather was sustained. We also saw in the T98G cell line over time (up to 6 h) a sustained PCM depolarization. This sustained PCM depolarization is an indication that upon apoptotic stimulation, the cells lose their ability to repolarize.

At this point it is important to mention the role of Bcl-2, the classic anti-apoptotic protein of the Bcl-2 family [72]. Usually, they are localized at the outer mitochondrial membrane, but recent studies discovered intracellular truncated forms in the neighborhood of the plasma cell membrane [73]. The most known function of Bcl-2 is exerted by inhibiting the oligomerization of Bcl-2-associated X protein (BAX) and Bcl-2-associated agonist of cell death protein (BAD) hereby preventing their pro-apoptotic effect.

But already decades ago the importance of Bcl-2 in modulating the plasma cell membrane has been stressed. We mentioned above the works of Gilbert and coworkers who observed that overexpression of the anti-apoptotic Bcl-2 gene is associated with membrane hyperpolarization rendering cells more resistant to radiation-induced apoptosis [61]. Further studies revealed that Bcl-2 itself has pore-forming domains similar to that of bacteria toxins and that the activation of K + channels by the myeloid leukemia cell differentiation protein (Mcl-1), a member of the Bcl-2 family, results in plasma cell membrane hyperpolarization [59, 74]. Finally, Lauf and coworkers could demonstrate a direct co-localization of Bcl-2 specifically with the NKA in the cell membrane providing the missing link to the hypothesized interaction between Bcl-2 and NKA [63].

The numerous functions of Ouabain on intracellular pathways are well described; one of them is the down-regulation of Bcl-2/Mcl-1 by accelerating its proteasomal degradation via reactive oxygen species (ROS) generation [75].

So, it seems, that Ouabain can not only depolarize the plasma cell membrane directly by inhibiting the NKA but additionally by down-regulating Bcl-2. We saw only in the TMZ-resistant T98G cell line a Bcl-2 down-regulation after treatment with Ouabain for 24 h at very low concentrations (0.01 nM) which unfortunately did not correlate with the concentrations, at which we observed PCM depolarization and cell migration inhibition. Consequently, in our study we could not confirm that Bcl-2 down-regulation contributes to PCM depolarization.

But with caution, we may interpret the down-regulation of Bcl-2 as a kind of sensitizing effect to facilitate e.g., apoptosis. Only recently, a Bcl-2 effect on cell migration was discovered [76–78]. In Fig. 1b we see a slight inhibitory effect of Ouabain on cell migration at very low (≤ 0.01 nM) concentrations which correlates exactly with those of Bcl-2 down-regulation (Fig. 5). As we outlined above, inhibition of cell migration is a prerequisite to apoptosis. Additional studies in future are needed and may focus not only on Bcl-2 but also on Mcl-1 expression [61, 63]. Interestingly, Wang and coworkers demonstrated that Mcl-1 causes a hyperpolarization of the PCMP through activation of K + channel activity [59] hereby preventing apoptosis.

At this point it should be stressed that Ouabain is known to have significant effects at nanomolar concentrations [56], e.g., at 0.1 to 10 nM the NKA is stimulated in non-malignant (cardiac and neuronal) cells, interestingly, via the high glycoside affinity α3 isoform [79].

As outlined above the PI3-K/Akt pathway is one important modulator of cell migration. We revealed in T98G cells an increase in p-Akt after 24 h treatment with 0.1 µM Ouabain. In contrast, LN229 cells did not show any change in p-Akt. It contradicts the anti-migratory effect of Ouabain which we revealed at 0.1 µM in T98G cells. In fact, we expected a p-Akt down-regulation, at least after prolonged 24 h treatment similar to Chen and coworkers, who observed a p-Akt down-regulation at 2.5 µM Ouabain in U-87 GBM cells [46].

The significant decreased level of pan-Akt after 24 h Ouabain treatment at 1 µM hints to a different mechanism by which Ouabain exerts its antitumor effects. You and coworkers were concerned about the short-lasting effects of phosphorylation inhibitors and developed a pan-AKT degrader by conjugating the Akt-phosphorylation inhibitor GDC-0068 to Lenalidomide. He showed that this compound (INY-03–041) induced significant degradation of all Akt isoforms at 24 h and, interestingly, improved the anti-proliferative effects compared to GDC-0068 alone [80].

Ouabain is known to contribute to degradation of several compounds in the signalosome by internalization and disturbed intracellular trafficking [81, 82]. With specific respect to the epidermal growth factor receptor (EGFR) Hafner and coworkers described in lung cancer A549 cells a specific phenomenon called endosomal arrest. After treatment with Ouabain, Digoxin, or Acovenoside they revealed persistent granules with internalized EGF-receptor without further degradation [83].

This endosomal arrest may be considered as a crucial checkpoint in cell biology [84] by diverting growth factors either to the recycling or the degradation pathway. In case of prolonged endosomal arrest, however, they are simply “stuck there”, losing any biological function.

We assume that pan-Akt like other compounds of the signalosome is endocytosed upon prolonged Ouabain stimulation and undergoes an endosomal arrest together with NKA α-subunits and EGFR. Indeed, it was shown that endosomal Akt is associated with the intracellular trafficking of growth factor receptor complexes and thus modifying their activity in a time and location dependent manner [85].

The peak in Akt activation (p-Akt Ser473) at prolonged lower doses of Ouabain might serve as a stimulus for inducing its degradation. Kometiani and coworkers stressed the time factor in intracellular activation processes i.e., while short term activation of ERK1/2 induced cell proliferation, sustained ERK1/2 activation resulted in increased expression of the cyclin-dependent kinase inhibitor 1 (p21Cip1) resulting in growth arrest [86]. Hence, this could be the main mechanism by which e.g., pan-Akt is down-regulated resulting in inhibition of migration and induction of apoptosis. Further studies certainly are here needed.

The reciprocal response of LN229 and T98G cells to Ouabain resp. Temozolomide is striking and may have significant clinical consequences. The underlying mechanisms are not yet known. There is evidence that Ouabain induces different endocytotic trafficking and signaling pathways according to the EGFR mutation status, the NKA isoforms [56, 87] and other not yet fully analyzed factors. Many authors described the interaction between NKA and EGFR e.g., Liu and Shapiro analyzed in the renal cell line LLC-PK1 the role of the signalosome in the process of endocytosis and demonstrated that Ouabain-stimulated endocytosis of the NKA is dependent on Caveolin-1 and Clathrin as well as the activation of c-Src, transactivation of EGFR and activation of phosphoinositide 3-kinase (PI3K). They showed that c-Src, EGFR, and the extracellular signal-regulated kinases 1 and 2 (ERK1/2) all were endocytosed along with the plasmalemmal NKA [81].

We did not analyze the NKA isoforms in our GBM cell lines but as shown by Chen and coworkers the T98G cell line is characterized by a high NKA α3/ α1 isoform ratio. He stressed that the high expression of the α3 isoform in the T98G cell line was correlating with a higher sensitivity to the apoptosis inducing effect of Ouabain [46]. Xiao and coworkers proved that the knockdown of the α3 isoform with siRNA impaired the anti-proliferative effect of Ouabain, indicating that Ouabain preferentially binds to the NKA α3 isoform [88]. Future studies are warranted to analyze the exact role of EGFR- and NKA isoform expression at the cell surface in directing ouabain-induced signaling either towards enhanced or reduced cell proliferation and migration.

Last, but not least, we could demonstrate for the first time an anti-angiogenic effect of Ouabain at low concentrations (0.01 µM) which correlated significantly with the inhibitory effect on cell migration (Fig. 7). Angiogenesis is considered as prerequisite for migration and invasion of tumor cells [89–91] and as such it constitutes an important target for cancer therapy, especially the hypervascularized gliomas. Bevacizumab a humanized monoclonal antibody against VEGF was approved in the treatment of recurrent GBM but, at least as monotherapy, it prolonged only the progression-free survival, but not the overall survival [92, 93]**.** The effect of Ouabain on angiogenesis resp. endothelial cells is rarely investigated [94, 95]**.** Trenti and coworkers revealed that Digitoxin in therapeutic range (1–25 nM) inhibited effectively angiogenesis via focal adhesion kinase (FAK) inhibition (51). At the same time Digitoxin as well as Ouabain protected HUVEC cells from apoptosis induced by growth factor deprivation (51). Dual actions of all CTS—dependent on cell types and dose regimen—remain a scientific and therapeutic challenge we have to accept and address in future studies.

## Conclusions and outlook

As described above, Ouabain induced in the TMZ-resistant T98G cell line inhibition of cell migration as well as depolarization of the plasma cell membrane both of which are preconditions for programmed cell death in malignant (here GBM) cells. Thus, a crucial, if not ubiquitous, phenomenon of this cardiotonic steroid is revealed.

We think that Ouabain is a promising new compound in the setting of TMZ-resistant/recurrent Glioblastoma multiforme and that it should be tested in combination with Temozolomide and irradiation in diverse in vitro and in vivo studies.

One limitation of our study was that we did not run Temozolomide as comparison in our experiments. Another general drawback is that significant higher Ouabain concentrations are used in experimental in vitro settings (0.1–10 µM) compared to those measured in vivo i.e., the therapeutic CTS range (1–50 nM) and endogenous CTS plasma concentrations (range 55–3000 pM) in humans. This might be due to differences between the cell environments and additional signaling factors in vivo, which may amplify or add to the Ouabain effect [96, 97]. An interesting approach would be to combine Ouabain with other phytochemicals shown to be active in GBM [98–100] and to deliver them with the recently investigated nanoparticle method [98, 101].

Moreover, further studies are needed and recommended to evaluate the exact mechanisms which are responsible for the resistance of T98G cells towards Temozolomide resp. their sensitivity towards Ouabain (e.g., MGMT status, their NKA isoform pattern, EGFR status, activation or degradation of growth factors like PI3-K, Akt, and the role of the intracellular milieu e.g., [Na]i/[K]i ratio and pH).

Finally, to improve the visualization of Ouabain-related pathways and the detection of endogenous Ouabain e.g., in tumor-bearing mice models we plan to develop a specific and sensitive aptamer-based diagnostic assay.

## Data Availability

The datasets for the current study are available from the corresponding author on request.

## References

[1] Albesiano E, Han JE, Lim M (2010). Mechanisms of local immunoresistance in glioma. Neurosurg Clin N Am.

[2] Le Rhun E, Rhun EL, Taillibert S, Chamberlain MC (2015). The future of high-grade glioma: where we are and where are we going. Surg Neurol Int.

[3] Gately L, McLachlan SA, Dowling A, Philip J (2017). Life beyond a diagnosis of glioblastoma: a systematic review of the literature. J Cancer Surviv.

[4] Hochberg FH, Pruitt A (1980). Assumptions in the radiotherapy of glioblastoma. Neurology.

[5] Bao S, Wu Q, McLendon RE, Hao Y, Shi Q, Hjelmeland AB (2006). Glioma stem cells promote radioresistance by preferential activation of the DNA damage response. Nature.

[6] Chargari C, Moncharmont C, Levy A, Guy JB, Bertrand G, Guilbert M (2012). Cancer stem cells, cornerstone of radioresistance and perspectives for radiosensitization: glioblastoma as an example. Bull Cancer.

[7] Peitzsch C, Perrin R, Hill RP, Dubrovska A, Kurth I (2014). Hypoxia as a biomarker for radioresistant cancer stem cells. Int J Radiat Biol.

[8] Stupp R, Mason WP, van den Bent MJ, Weller M, Fisher B, Taphoorn MJ (2005). Radiotherapy plus concomitant and adjuvant temozolomide for glioblastoma. N Engl J Med.

[9] Stupp R, Hegi ME, Mason WP, van den Bent MJ, Taphoorn MJ, Janzer RC (2009). Effects of radiotherapy with concomitant and adjuvant temozolomide versus radiotherapy alone on survival in glioblastoma in a randomised phase III study: 5-year analysis of the EORTC-NCIC trial. Lancet Oncol.

[10] Hasselbalch B, Lassen U, Hansen S, Holmberg M, Sorensen M, Kosteljanetz M (2010). Cetuximab, bevacizumab, and irinotecan for patients with primary glioblastoma and progression after radiation therapy and temozolomide: a phase II trial. Neuro Oncol.

[11] Jiang P, Mukthavaram R, Chao Y, Bharati IS, Fogal V, Pastorino S (2014). Novel anti-glioblastoma agents and therapeutic combinations identified from a collection of FDA approved drugs. J Transl Med.

[12] Olson JJ, Nayak L, Ormond DR, Wen PY, Kalkanis SN, Committee ACJG (2014). The role of cytotoxic chemotherapy in the management of progressive glioblastoma : a systematic review and evidence-based clinical practice guideline. J Neurooncol.

[13] Martinho O, Silva-Oliveira R, Miranda-Goncalves V, Clara C, Almeida JR, Carvalho AL (2013). In vitro and in vivo analysis of RTK inhibitor efficacy and identification of its novel targets in glioblastomas. Transl Oncol.

[14] Padfield E, Ellis HP, Kurian KM (2015). Current therapeutic advances targeting EGFR and EGFRvIII in Glioblastoma. Front Oncol.

[15] Wachsberger PR, Lawrence YR, Liu Y, Daroczi B, Xu X, Dicker AP (2012). Epidermal growth factor receptor expression modulates antitumor efficacy of vandetanib or cediranib combined with radiotherapy in human glioblastoma xenografts. Int J Radiat Oncol Biol Phys.

[16] Beal K, Abrey LE, Gutin PH (2011). Antiangiogenic agents in the treatment of recurrent or newly diagnosed glioblastoma: analysis of single-agent and combined modality approaches. Radiat Oncol.

[17] Lu-Emerson C, Duda DG, Emblem KE, Taylor JW, Gerstner ER, Loeffler JS (2015). Lessons from anti-vascular endothelial growth factor and anti-vascular endothelial growth factor receptor trials in patients with glioblastoma. J Clin Oncol.

[18] Messaoudi K, Clavreul A, Lagarce F (2015). Toward an effective strategy in glioblastoma treatment. Part I: resistance mechanisms and strategies to overcome resistance of glioblastoma to temozolomide. Drug Discov Today.

[19] Sanati M, Binabaj MM, Ahmadi SS, Aminyavari S, Javid H, Mollazadeh H (2022). Recent advances in glioblastoma multiforme therapy: a focus on autophagy regulation. Biomed Pharmacother.

[20] Suryadevara CM, Verla T, Sanchez-Perez L, Reap EA, Choi BD, Fecci PE (2015). Immunotherapy for malignant glioma. Surg Neurol Int.

[21] Chiarelli PA, Kievit FM, Zhang M, Ellenbogen RG (2015). Bionanotechnology and the future of glioma. Surg Neurol Int.

[22] Doris PA, Hayward-Lester A, Bourne D, Stocco DM (1996). Ouabain production by cultured adrenal cells. Endocrinology.

[23] Hamlyn JM, Blaustein MP, Bova S, DuCharme DW, Harris DW, Mandel F (1991). Identification and characterization of a ouabain-like compound from human plasma. Proc Natl Acad Sci U S A.

[24] Hamlyn JM, Lu ZR, Manunta P, Ludens JH, Kimura K, Shah JR (1998). Observations on the nature, biosynthesis, secretion and significance of endogenous ouabain. Clin Exp Hypertens.

[25] Laredo J, Shah JR, Lu ZR, Hamilton BP, Hamlyn JM (1997). Angiotensin II stimulates secretion of endogenous ouabain from bovine adrenocortical cells via angiotensin type 2 receptors. Hypertension.

[26] Sophocleous A, Elmatzoglou I, Souvatzoglou A (2003). Circulating endogenous digitalis-like factor(s) (EDLF) in man is derived from the adrenals and its secretion is ACTH-dependent. J Endocrinol Invest.

[27] Baecher S, Kroiss M, Fassnacht M, Vogeser M (2014). No endogenous ouabain is detectable in human plasma by ultra-sensitive UPLC-MS/MS. Clin Chim Acta.

[28] Lewis LK, Yandle TG, Hilton PJ, Jensen BP, Begg EJ, Nicholls MG (2014). Endogenous ouabain is not ouabain. Hypertension.

[29] Kiani Z, Shafiei M, Rahimi-Moghaddam P, Karkhane AA, Ebrahimi SA (2012). In vitro selection and characterization of deoxyribonucleic acid aptamers for digoxin. Anal Chim Acta.

[30] Pfeiffer F, Mayer G (2016). Selection and biosensor application of aptamers for small molecules. Front Chem.

[31] Tokhtaeva E, Clifford RJ, Kaplan JH, Sachs G, Vagin O (2012). Subunit isoform selectivity in assembly of Na K-ATPase alpha-beta heterodimers. J Biol Chem.

[32] Mobasheri A, Fox R, Evans I, Cullingham F, Martin-Vasallo P, Foster CS (2003). Epithelial Na, K-ATPase expression is down-regulated in canine prostate cancer; a possible consequence of metabolic transformation in the process of prostate malignancy. Cancer Cell Int.

[33] Sakai H, Suzuki T, Maeda M, Takahashi Y, Horikawa N, Minamimura T (2004). Up-regulation of Na(+), K(+)-ATPase alpha 3-isoform and down-regulation of the alpha1-isoform in human colorectal cancer. FEBS Lett.

[34] Shibuya K, Fukuoka J, Fujii T, Shimoda E, Shimizu T, Sakai H (2010). Increase in ouabain-sensitive K+-ATPase activity in hepatocellular carcinoma by overexpression of Na+, K+-ATPase alpha 3-isoform. Eur J Pharmacol.

[35] Newman RA, Yang P, Pawlus AD, Block KI (2008). Cardiac glycosides as novel cancer therapeutic agents. Mol Interv.

[36] Aizman O, Aperia A (2003). Na, K-ATPase as a signal transducer. Ann N Y Acad Sci.

[37] Haas M, Wang H, Tian J, Xie Z (2002). Src-mediated inter-receptor cross-talk between the Na+/K+-ATPase and the epidermal growth factor receptor relays the signal from ouabain to mitogen-activated protein kinases. J Biol Chem.

[38] Xie Z (2001). Ouabain interaction with cardiac Na/K-ATPase reveals that the enzyme can act as a pump and as a signal transducer. Cell Mol Biol (Noisy-le-grand).

[39] Shiratori O (1967). Growth inhibitory effect of cardiac glycosides and aglycones on neoplastic cells: in vitro and in vivo studies. Gan.

[40] Al-Ghoul M, Valdes R (2008). Mammalian cardenolides in cancer prevention and therapeutics. Ther Drug Monit.

[41] Chen JQ, Contreras RG, Wang R, Fernandez SV, Shoshani L, Russo IH (2006). Sodium/potassium ATPase (Na+, K+-ATPase) and ouabain/related cardiac glycosides: a new paradigm for development of anti- breast cancer drugs?. Breast Cancer Res Treat.

[42] Haux J (1999). Digitoxin is a potential anticancer agent for several types of cancer. Med Hypotheses.

[43] Lopez-Lazaro M (2007). Digitoxin as an anticancer agent with selectivity for cancer cells: possible mechanisms involved. Expert Opin Ther Targets.

[44] Badr CE, Wurdinger T, Tannous BA (2011). Functional drug screening assay reveals potential glioma therapeutics. Assay Drug Dev Technol.

[45] Denicolai E, Baeza-Kallee N, Tchoghandjian A, Carre M, Colin C, Jiglaire CJ (2014). Proscillaridin A is cytotoxic for glioblastoma cell lines and controls tumor xenograft growth in vivo. Oncotarget.

[46] Chen D, Song M, Mohamad O, Yu SP (2014). Inhibition of Na+/K+-ATPase induces hybrid cell death and enhanced sensitivity to chemotherapy in human glioblastoma cells. BMC Cancer.

[47] Yang XS, Xu ZW, Yi TL, Xu RC, Li J, Zhang WB (2018). Ouabain suppresses the growth and migration abilities of glioma U87MG cells through inhibiting the Akt/mTOR signaling pathway and downregulating the expression of HIF1alpha. Mol Med Rep.

[48] Lefranc F, Kiss R (2008). The sodium pump alpha1 subunit as a potential target to combat apoptosis-resistant glioblastomas. Neoplasia.

[49] Menger L, Vacchelli E, Kepp O, Eggermont A, Tartour E, Zitvogel L (2013). Trial watch: Cardiac glycosides and cancer therapy. Oncoimmunology.

[50] Taylor MA, Das BC, Ray SK (2018). Targeting autophagy for combating chemoresistance and radioresistance in glioblastoma. Apoptosis.

[51] Goldberger ZD, Goldberger AL (2012). Therapeutic ranges of serum digoxin concentrations in patients with heart failure. Am J Cardiol.

[52] Trenti A, Zulato E, Pasqualini L, Indraccolo S, Bolego C, Trevisi L (2017). Therapeutic concentrations of digitoxin inhibit endothelial focal adhesion kinase and angiogenesis induced by different growth factors. Br J Pharmacol.

[53] Harwood S, Little JA, Gallacher G, Perrett D, Edwards R, Dawnay A (1997). Development of enzyme immunoassay for endogenous ouabain-like compound in human plasma. Clin Chem.

[54] Lopatin DA, Ailamazian EK, Dmitrieva RI, Shpen VM, Fedorova OV, Doris PA (1999). Circulating bufodienolide and cardenolide sodium pump inhibitors in preeclampsia. J Hypertens.

[55] Manunta P, Stella P, Rivera R, Ciurlino D, Cusi D, Ferrandi M (1999). Left ventricular mass, stroke volume, and ouabain-like factor in essential hypertension. Hypertension.

[56] Weidemann H (2012). "The Lower Threshold" phenomenon in tumor cells toward endogenous digitalis-like compounds: Responsible for tumorigenesis?. J Carcinog.

[57] Litan A, Langhans SA (2015). Cancer as a channelopathy: ion channels and pumps in tumor development and progression. Front Cell Neurosci.

[58] Molenaar RJ (2011). Ion channels in glioblastoma. ISRN Neurol.

[59] Wang L, Zhou P, Craig RW, Lu L (1999). Protection from cell death by mcl-1 is mediated by membrane hyperpolarization induced by K(+) channel activation. J Membr Biol.

[60] Chittajallu R, Chen Y, Wang H, Yuan X, Ghiani CA, Heckman T (2002). Regulation of Kv1 subunit expression in oligodendrocyte progenitor cells and their role in G1/S phase progression of the cell cycle. Proc Natl Acad Sci U S A.

[61] Gilbert MS, Saad AH, Rupnow BA, Knox SJ (1996). Association of BCL-2 with membrane hyperpolarization and radioresistance. J Cell Physiol.

[62] Gilbert M, Knox S (1997). Influence of Bcl-2 overexpression on Na+/K(+)-ATPase pump activity: correlation with radiation-induced programmed cell death. J Cell Physiol.

[63] Lauf PK, Alqahtani T, Flues K, Meller J, Adragna NC (2015). Interaction between Na-K-ATPase and Bcl-2 proteins BclXL and Bak. Am J Physiol Cell Physiol.

[64] Vultur A, Buettner R, Kowolik C, Liang W, Smith D, Boschelli F (2008). SKI-606 (bosutinib), a novel Src kinase inhibitor, suppresses migration and invasion of human breast cancer cells. Mol Cancer Ther.

[65] Heiss M, Hellstrom M, Kalen M, May T, Weber H, Hecker M (2015). Endothelial cell spheroids as a versatile tool to study angiogenesis in vitro. FASEB J.

[66] Korff T, Augustin HG (1999). Tensional forces in fibrillar extracellular matrices control directional capillary sprouting. J Cell Sci.

[67] Korff T, Augustin HG (1998). Integration of endothelial cells in multicellular spheroids prevents apoptosis and induces differentiation. J Cell Biol.

[68] Joy AM, Beaudry CE, Tran NL, Ponce FA, Holz DR, Demuth T (2003). Migrating glioma cells activate the PI3-K pathway and display decreased susceptibility to apoptosis. J Cell Sci.

[69] Lefranc F, Brotchi J, Kiss R (2005). Possible future issues in the treatment of glioblastomas: special emphasis on cell migration and the resistance of migrating glioblastoma cells to apoptosis. J Clin Oncol.

[70] Suzuki-Karasaki Y, Suzuki-Karasaki M, Uchida M, Ochiai T (2014). Depolarization controls TRAIL-sensitization and tumor-selective killing of cancer cells: crosstalk with ROS. Front Oncol.

[71] Bortner CD, Gomez-Angelats M, Cidlowski JA (2001). Plasma membrane depolarization without repolarization is an early molecular event in anti-Fas-induced apoptosis. J Biol Chem.

[72] Hatok J, Racay P (2016). Bcl-2 family proteins: master regulators of cell survival. Biomol Concepts.

[73] Borner C, Martinou I, Mattmann C, Irmler M, Schaerer E, Martinou JC (1994). The protein bcl-2 alpha does not require membrane attachment, but two conserved domains to suppress apoptosis. J Cell Biol.

[74] Schendel SL, Xie Z, Montal MO, Matsuyama S, Montal M, Reed JC (1997). Channel formation by antiapoptotic protein Bcl-2. Proc Natl Acad Sci U S A.

[75] Chanvorachote P, Pongrakhananon V (2013). Ouabain downregulates Mcl-1 and sensitizes lung cancer cells to TRAIL-induced apoptosis. Am J Physiol Cell Physiol.

[76] Fouque A, Lepvrier E, Debure L, Gouriou Y, Malleter M, Delcroix V (2016). The apoptotic members CD95, BclxL, and Bcl-2 cooperate to promote cell migration by inducing Ca(2+) flux from the endoplasmic reticulum to mitochondria. Cell Death Differ.

[77] Koehler BC, Scherr AL, Lorenz S, Urbanik T, Kautz N, Elssner C (2013). Beyond cell death - antiapoptotic Bcl-2 proteins regulate migration and invasion of colorectal cancer cells in vitro. PLoS ONE.

[78] Um HD (2016). Bcl-2 family proteins as regulators of cancer cell invasion and metastasis: a review focusing on mitochondrial respiration and reactive oxygen species. Oncotarget.

[79] Gao J, Wymore RS, Wang Y, Gaudette GR, Krukenkamp IB, Cohen IS (2002). Isoform-specific stimulation of cardiac Na/K pumps by nanomolar concentrations of glycosides. J Gen Physiol.

[80] You I, Erickson EC, Donovan KA, Eleuteri NA, Fischer ES, Gray NS (2020). Discovery of an AKT degrader with prolonged inhibition of downstream signaling. Cell Chem Biol.

[81] Liu J, Shapiro JI (2007). Regulation of sodium pump endocytosis by cardiotonic steroids: molecular mechanisms and physiological implications. Pathophysiology.

[82] Rosen H, Glukhman V, Feldmann T, Fridman E, Lichtstein D (2004). Cardiac steroids induce changes in recycling of the plasma membrane in human NT2 cells. Mol Biol Cell.

[83] Hafner S, Schmiech M, Lang SJ (2021). The cardenolide glycoside acovenoside a interferes with epidermal growth factor receptor trafficking in non-small cell lung cancer cells. Front Pharmacol.

[84] Cota CD, Dreier MS, Colgan W, Cha A, Sia T, Davidson B (2021). Cyclin-dependent Kinase 1 and Aurora Kinase choreograph mitotic storage and redistribution of a growth factor receptor. PLoS Biol.

[85] Szymonowicz K, Oeck S, Malewicz NM, Jendrossek V (2018). New insights into protein kinase B/Akt signaling: role of localized Akt activation and compartment-specific target proteins for the cellular radiation response. Cancers (Basel).

[86] Kometiani P, Liu L, Askari A (2005). Digitalis-induced signaling by Na+/K+-ATPase in human breast cancer cells. Mol Pharmacol.

[87] Mijatovic T, Op De Beeck A, Van Quaquebeke E, Dewelle J, Darro F, de Launoit Y (2006). The cardenolide UNBS1450 is able to deactivate nuclear factor kappaB-mediated cytoprotective effects in human non-small cell lung cancer cells. Mol Cancer Ther.

[88] Xiao Y, Meng C, Lin J, Huang C, Zhang X, Long Y (2017). Ouabain targets the Na(+)/K(+)-ATPase alpha3 isoform to inhibit cancer cell proliferation and induce apoptosis. Oncol Lett.

[89] Ahir BK, Engelhard HH, Lakka SS (2020). Tumor development and angiogenesis in adult brain tumor: glioblastoma. Mol Neurobiol.

[90] Mosteiro A, Pedrosa L, Ferres A, Diao D, Sierra A, Gonzalez JJ (2022). The vascular microenvironment in glioblastoma: a comprehensive review. Biomedicines.

[91] Zhang M, Ye G, Li J, Wang Y (2015). Recent advance in molecular angiogenesis in glioblastoma: the challenge and hope for anti-angiogenic therapy. Brain Tumor Pathol.

[92] Kim MM, Umemura Y, Leung D (2018). Bevacizumab and glioblastoma: past, present, and future directions. Cancer J.

[93] Zhang T, Xin Q, Kang JM (2021). Bevacizumab for recurrent glioblastoma: a systematic review and meta-analysis. Eur Rev Med Pharmacol Sci.

[94] Nagaoka K, Kurauchi Y, Asano D, Morita A, Sakamoto K, Nakahara T (2022). Pharmacological inhibition of Na(+)/K(+)-ATPase induces neurovascular degeneration and glial cell alteration in the rat retina. Exp Eye Res.

[95] Zhao GH, Qiu YQ, Yang CW, Chen IS, Chen CY, Lee SJ (2020). The cardenolides ouabain and reevesioside A promote FGF2 secretion and subsequent FGFR1 phosphorylation via converged ERK1/2 activation. Biochem Pharmacol.

[96] Jansson-Lofmark R, Hjorth S, Gabrielsson J (2020). Does in vitro potency predict clinically efficacious concentrations?. Clin Pharmacol Ther.

[97] Poole-Wilson PA, Galindez E, Fry CH (1979). Effect of ouabain in therapeutic concentrations on K+ exchange and contraction of human and rabbit myocardium. Clin Sci (Lond).

[98] Cao H, Li X, Wang F, Zhang Y, Xiong Y, Yang Q (2020). Phytochemical-mediated glioma targeted treatment: drug resistance and novel delivery systems. Curr Med Chem.

[99] Mohamadian M, Ahmadi SS, Bahrami A, Ferns GA (2022). Review on the therapeutic potential of curcumin and its derivatives on glioma biology. Neurochem Res.

[100] Qoorchi Moheb Seraj F, Heravi-Faz N, Soltani A, Ahmadi SS, Shahbeiki F, Talebpour A (2022). Thymol has anticancer effects in U-87 human malignant glioblastoma cells. Mol Biol Rep.

[101] Sanati M, Afshari AR, Kesharwani P, Sukhorukov VN, Sahebkar A (2022). Recent trends in the application of nanoparticles in cancer therapy: the involvement of oxidative stress. J Control Release.

